# Impact of estroprogestin therapies on bone health from adolescence to postmenopause

**DOI:** 10.1007/s40618-025-02802-2

**Published:** 2026-02-18

**Authors:** Guabello G, Federici S, Carrara S, Ruotolo E, Rossi A, Bertini J, Bonomi M, Grandi G, Corbetta S

**Affiliations:** 1 Division of Internal Medicine, Endocrinology and Metabolic Diseases, I.R.C.C.S. Ospedale Galeazzi – Sant’Ambrogio, Milan, Italy; 2 Department of Endocrine and Metabolic Diseases, I.R.C.C.S. Istituto Auxologico Italiano, Milan, Italy; 3 Department of Medical Biotechnology and Translational Medicine, University of Milan, Milan, Italy; 4 Department of Biomedical and Clinical Sciences, University of Milan, Milan, Italy; 5 Department of Medical and Surgical Sciences for Mother, Child and Adult, University of Modena and Reggio Emilia, Modena, Italy; 6 Azienda Ospedaliero Universitaria, Modena, Italy; 7 Bone Metabolism Diseases and Diabetes Unit, IRCCS Istituto Auxologico Italiano, Via L. Ariosto 9, 20145 Milan, Italy; 8 Department of Biomedical, Surgical and Dental Sciences, University of Milan, Milan, Italy

**Keywords:** Estrogens, Progestins, Bone remodelling, Osteoporosis

## Abstract

**Background:**

Estrogens are fundamental to bone health, exerting life-long effects onbone metabolism. They regulate skeletal growth and maturation during adolescence andmaintain bone remodeling in adulthood. The decline in estrogen levels during perimenopauseand postmenopause leads to bone mass loss. Emerging evidence also supports a role forprogestins in bone metabolism. Several estrogen-progestins formulations are available andwidely used for contraception in fertile women and as hormone replacement therapy (HRT) incases of primary or secondary ovarian insufficiency as well as in physiologic menopause.Advances in both synthetic and natural preparations allowed for more personalized treatmentapproaches. When selecting estrogen-progestin therapies, it is essential to consider their impacton bone health and remodelling.

**Objective:**

This narrative review examines the effects of variousestrogen-progestin regimens on bone metabolism across the female lifespan, focusing on mechanisms of action, safety profiles, and clinical applications. Here we show that combined oralcontraceptives (COCs) are generally safe for bone mineral density (BMD) in adult women;however, caution is advised in adolescence, especially with very low-dose estrogen formulations.In hypogonadal women, postmenopausal hormone replacement therapy (HRT) is key forosteoporosis prevention. Physiological regimens, preferably transdermal estrogen at doseshigher than those used in typical postmenopausal therapy, may offer better bone protection.Additionally, oral gonadotropin-releasing hormone receptor (GnRH-R) antagonists combinedwith add-back therapy represent a novel long-term option for managing uterine fibroids andendometriosis without compromising BMD. In postmenopausal women, both HRT and selectiveestrogen receptor modulators (SERMs) effectively reduce fracture risk and support bone health.

**Main results & implications:**

Optimizing therapeutic strategies to balance efficacy and safety, iscritical to ensuring personalized care that preserves bone health and enhances quality of lifeacross a woman’s lifespan.

## Introduction

Estrogen-progestin therapies have a significant impact on bone health from adolescence to postmenopause. The physiological role of estrogens in bone metabolism is well-documented: they regulate growth and skeletal maturation during adolescence, sustain bone remodelling in adulthood, and mitigate the effects of bone loss during perimenopause and postmenopause. However, hormonal imbalances or deficiencies during key life stages, such as in reproductive-age women with hypogonadism or physiological menopause, can accelerate bone loss and increase the risk of osteoporosis and fractures.

Advances in estrogen and estrogen-progestin therapies, including synthetic and natural formulations, offer tailored solutions to address specific clinical needs. In addition, innovative treatments, such as oral gonadotropin-releasing hormone receptor antagonists (relugolix, linzagolix) with add-back therapy, provide effective non-surgical options for conditions like uterine fibroids and endometriosis, while preserving bone health.

It is crucial to consider the bone status when considering the choice of the different estrogen-progestin therapy; besides, it’s important to be aware about the effects of the different preparations on bone remodeling.

This narrative review examines the effects of various estrogen-progestin therapies on bone metabolism across lifespan, focusing on their mechanisms of action, safety profiles, and clinical applications to guide personalized and effective therapeutic strategies. Of note, the majority of studies investigated bone mineral density and bone turnover parameters, while data about bone quality assessment are very limited.

## Effects of estrogens on bone tissue

Estrogens influence bone tissue at different stages of a woman’s life through varied and complex mechanisms.

**During adolescence**, estrogens are pivotal for the pubertal growth spurt by activating the growth hormone (GH)-IGF-I axis, promoting periosteal apposition, and inducing closure of the growth plates, thus ending growth earlier in females than in males due to higher estrogen levels. Before puberty, skeletal features show no significant gender differences; in this phase, the GH/IGF-1 axis induce a slow, but continuous, bone growth. At the onset of puberty, the low estrogens concentration stimulates the growth spurt and the longitudinal growth via indirect effects on growth hormone (GH) and insulin-like growth factor I (IGF-I), which both stimulate growth plate chondrocytes. During the late stages of puberty, the higher estrogen concentrations stimulate the closure of the epiphyseal growth plates [1]. Approximately 90% of peak bone mass is achieved by the age of 18 [2].

Limited data are available about bone strength accrual during growth and adaptations in bone microstructure, density, and geometry that accompany gains in bone strength. A high resolution- Peripheral Quantitative Computed Tomography (HR-pQCT) study at the distal tibia and radius in 184 boys and 209 girls (aged 9–20 years at baseline) demonstrate, across 12 years of longitudinal growth, a boys’ superior bone size and strength compared with girls that may confer them a protective advantage [3]. However, boys’ consistently more porous cortices may contribute to boys’ higher fracture incidence during adolescence.

**Fig. 1:**
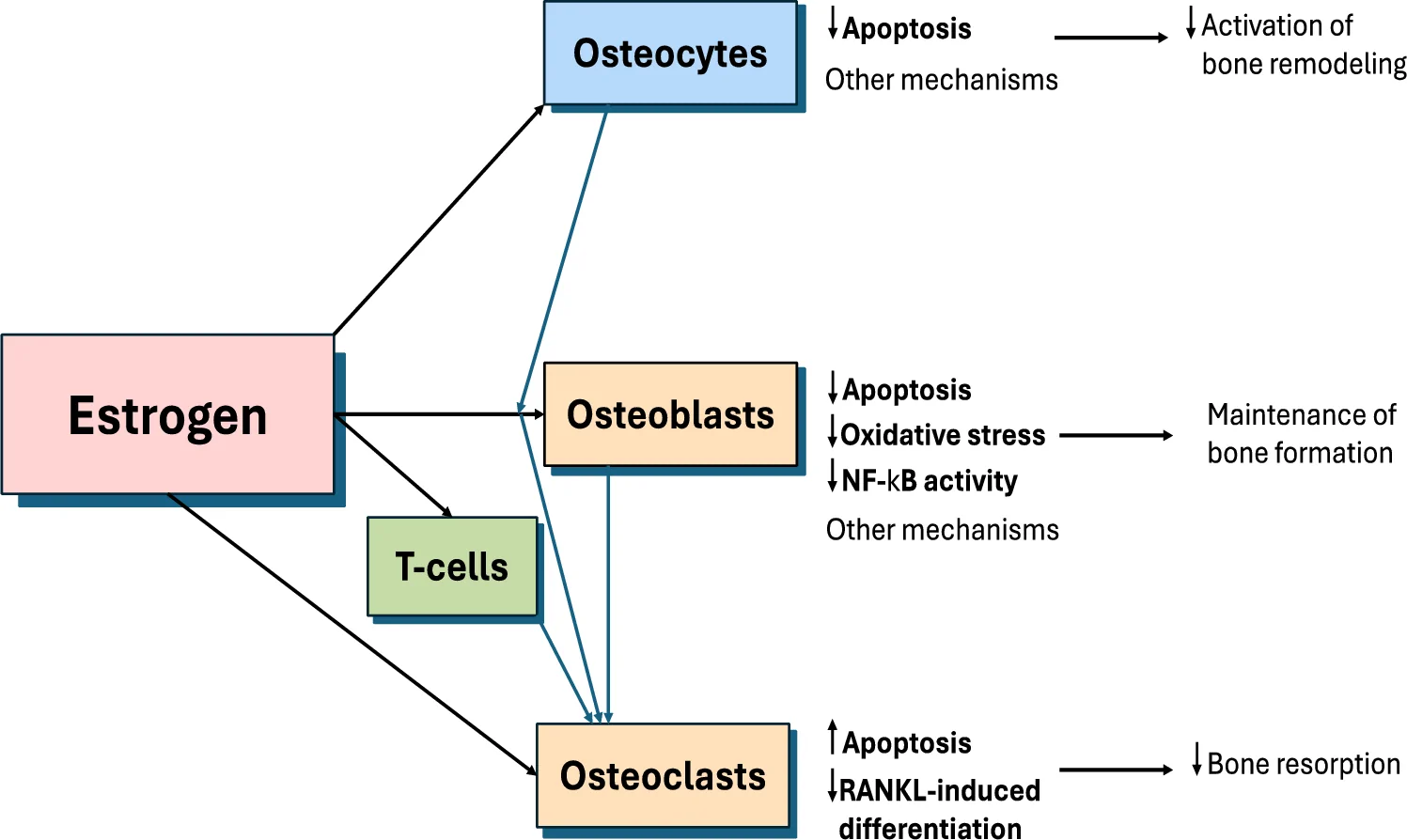
Actions of estrogens in osteoblasts, osteocytes and osteoclasts; the immune T-cells are also considered. NF-kB, nuclear factor k-light-chain-enhancer of activated B cells

**In adulthood**, estrogens exert their effects on bone tissue primarily through an agonist binding to cytoplasmic estrogen receptor alpha (ERα), leading to nuclear genomic transcription. In osteoblasts (bone matrix-depositing cells) estrogens enhance the release of osteoprotegerin (a "decoy" receptor for RANKL), thereby inhibiting osteoclast activation and reducing osteoblast apoptosis. In osteocytes, estrogens decrease the production of sclerostin (a potent inhibitor of osteoblastogenesis) and also reduce osteocyte apoptosis, collectively promoting bone formation and preserving bone mass (Fig. 1).

**Fig. 2:**
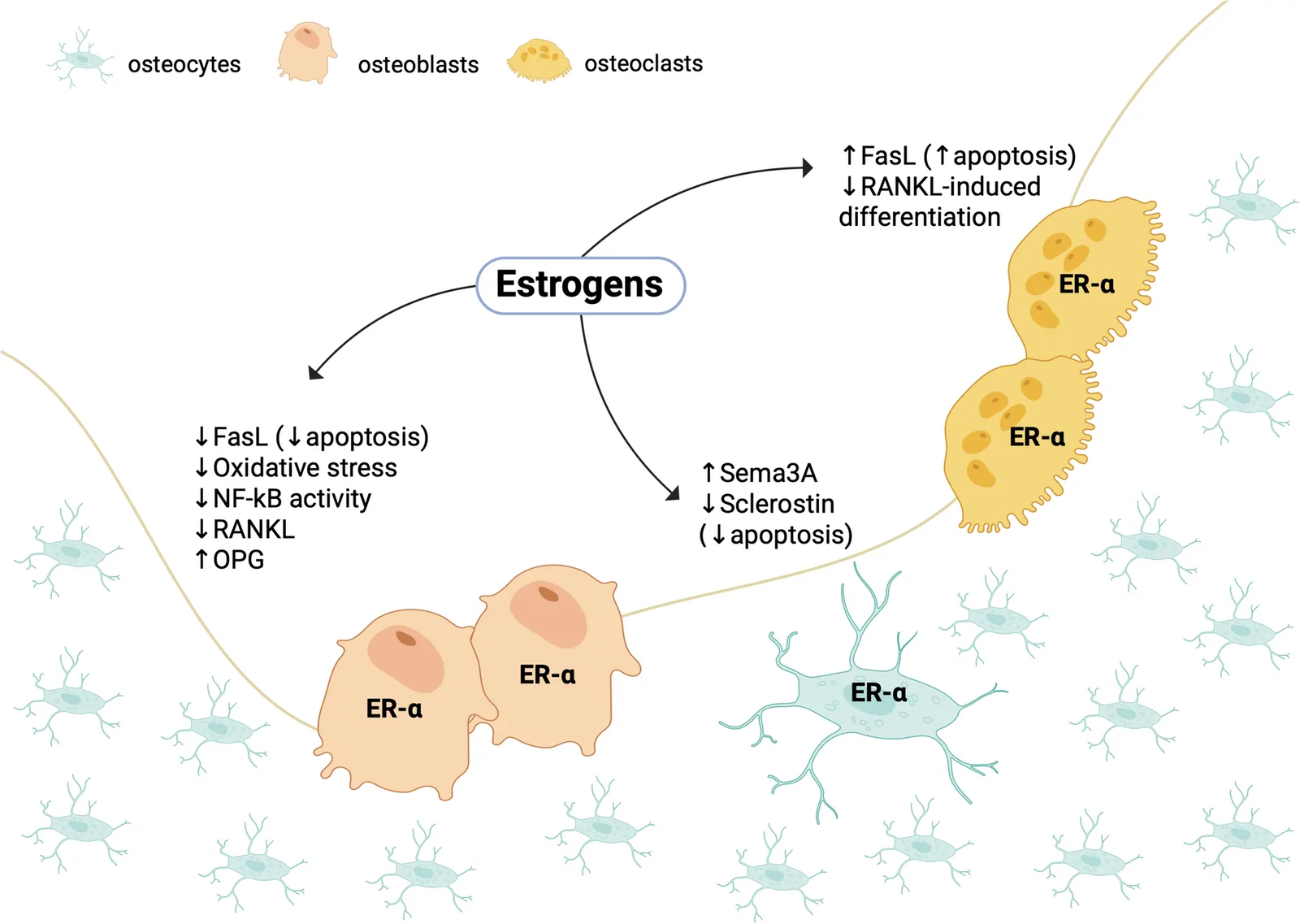
Bone cells targets and cell-specific effects of estrogens. ER-a, estrogen receptor-a; FasL, Fas ligand [a transmembrane protein member of the tumour necrosis factor (TNF) family which interacts with the Fas (CD95; APO-1; TNFRSF6) receptor]; NF-kB, nuclear factor kappa-light-chain-enhancer of activated B cells; RANKL, Receptor activator of nuclear factor kappa-Β ligand; OPG, osteoprotegerin; Sema3A, semaphorin 3A

Estrogens act on the osteoblast–osteoclast cross-talk by stimulating osteoblasts to release Semaphorin 3 A, a cytokine with osteoprotegerin-like activity and therefore anti-osteoclastogenic properties. *In vivo* studies in mice demonstrated that loss of Sema3A induces severe osteopenia both in trabecular and cortical bones due to increased numbers of osteoclasts and decreased numbers of osteoblasts [4] (Fig. 2).

Additionally, estrogens impact immune cells by inhibiting the release of TNF-α, IL-1, IL-6, and IL-7 (potent stimulators of bone resorption) and provide oxidative stress protection, counteracting osteoblast aging. Through these mechanisms, estrogens exert a trophic effect on bone tissue, maintaining low turnover during a woman’s reproductive years [5].

**Fig. 3:**
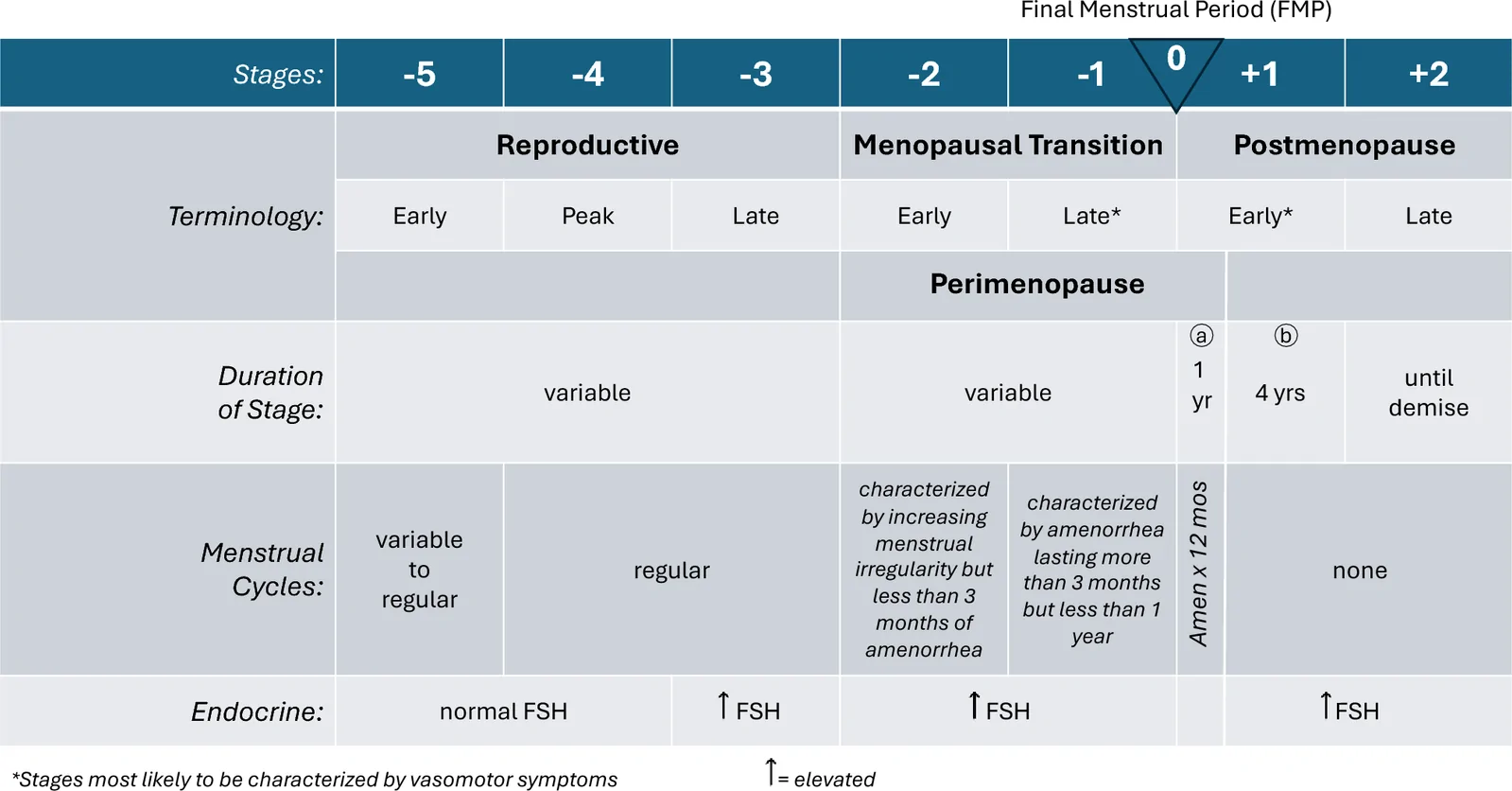
Early and late stages of perimenopause according to the Reproductive Aging Workshop (STRAW +10) criteria. Adapted from (5). Yr, year; yrs, years; amen, amenorrhea; mos, months; FSH, follicle stimulating hormone; ↑, increased

**Perimenopause** represents a transitional phase in a woman's life, divided into an "early" phase, characterized by increasing menstrual irregularity and less than 3 months of amenorrhea, and a "late" phase, marked by more than 3 months but less than 1 year of amenorrhea. Menopause is defined after at least 12 consecutive months of amenorrhea. Bone loss begins during the "late" perimenopause and accelerates in menopause, where significantly low estrogens levels result in marked alterations in the osteoblast-osteoclast crosstalk, increased bone resorption, elevated pro-osteoclastic cytokines, and reduced oxidative stress defences for osteoblasts [6, 7] (Fig. 3).

## Circulating estradiol levels and bone health

Under physiological conditions, estradiol levels vary across the different phases of the menstrual cycle. During the follicular phase, estradiol levels progressively rise, ranging between 50 and 150 pg/mL, peaking at 330–700 pg/mL during ovulation, then sharply decline and stabilize at approximately 200 pg/mL during the mid-luteal phase. According to *estrogen threshold hypothesis,* tissue sensitivity to estradiol varies, and it is proposed that during a woman’s fertile years estradiol levels between 30 and 60 pg/mL are sufficient to prevent bone mass loss. Below a threshold of 30 pg/mL, there is progressive bone mineral density (BMD) loss and bone depletion [8]. However, the ultimate validity of this threshold has not been demonstrated and does not account for inter-individual variability [9].

During menopause, estradiol levels decline significantly but do not fall to zero as peripheral aromatization of testosterone continues to produce small amount of estrogen. Levels stabilize between 10 and 38 pg/mL. Studies demonstrate that postmenopausal women under 65 years of age with estradiol levels above 10 pg/mL have significantly higher lumbar and femoral BMD compared to women with estradiol levels below 10 pg/mL [10].

## Natural and synthetic estrogens

Estrogens used in estrogenic and estrogen-progestin therapeutic formulations are categorized into natural and synthetic estrogens. *Natural estrogens* can be either human-derived (17β-estradiol [E2], estradiol valerate [E2V], estetrol [E4]) or animal-derived (conjugated equine estrogens [CEE]). The only *synthetic estrogen* is 17α-ethinyl estradiol (EE).

**17β-Estradiol (E2)** is the most potent natural estrogen. When administered orally, it is metabolized in the liver into physiological molecules such as estrone (E1) and estriol (E3), which exert estrogenic effects on all estrogen-sensitive organs, including the central nervous system. Compared to 17α-ethinyl estradiol, E2 has a lesser impact on liver estrogen-dependent markers (SHBG, HDL, VLDL, angiotensinogen) during first-pass metabolism. When administered transdermally, first-pass hepatic metabolism is bypassed entirely [11].

**Estradiol Valerate (E2V)** is the esterified form of 17β-estradiol. Administered orally, it is rapidly and completely metabolized to E2 during first-pass hepatic metabolism (1 mg E2V = 0.76 mg E2). It has nearly identical pharmacokinetics and identical pharmacodynamics to estradiol [12, 13]. Both 17β-estradiol and estradiol valerate are included in certain formulations of hormone replacement therapy (HRT) and combined oral contraceptives (COCs), such as estradiol/nomegestrol acetate and estradiol valerate/dienogest, available since 2010.

**Estetrol (E4)** is a natural estrogen produced by the fetal liver beginning around the ninth week of gestation, which then enters the maternal circulation through the placenta. Maternal blood levels of estetrol during pregnancy range from 400 to 1,200 pg/mL, with fetal levels approximately ten times higher. Its physiological role remains unknown. Estetrol is classified as a Natural Estrogen with Selective Tissue Activity (NEST). Unlike estradiol, which acts as an agonist on both the membrane receptor GPER1 (mediating rapid non-genomic effects) and the cytoplasmic estrogen receptor alpha (mediating slower genomic transcription effects), estetrol functions as an agonist on the cytoplasmic receptor but as an antagonist on GPER1. This agonist/antagonist activity underlies its beneficial effects on vaginal epithelium, endometrium, bone tissue, and the cardiovascular system, with minimal hepatic impact on hemostatic and lipid profiles and a negligible or neutral effect on breast tissue [14–16]. Estetrol has been included in a COC (estetrol/drospirenone), available since 2021.

**Conjugated Equine Estrogens (CEE)** (e.g., estrone sulfate, equilin sulfate, 17α-dihydroequilin sulfate) are animal-derived (obtained from the urine of pregnant mares). Administered orally, these estrogens recirculate in the bloodstream until degraded and excreted in urine, without undergoing metabolic conversion (as they are not recognized by the body as physiological steroids) [17]. CEEs are components of certain HRT formulations, both historical and recently introduced.

**17α-Ethinyl Estradiol (EE)** is a synthetic estrogen created by adding an ethinyl group to estradiol. This modification produces a compound administered orally in very low doses (micrograms) with significantly higher potency than natural estrogens. Even in small doses, EE suppresses ovarian activity when combined with a progestin with strong anti-gonadotropic activity. The ethinyl group prevents the molecule’s inactivation and contributes to its slow metabolism (half-life of 13–27 hours). Compared to natural estradiol, EE exerts a greater influence on liver estrogen-dependent markers (SHBG, HDL, VLDL, angiotensinogen) during first-pass hepatic metabolism. This effect, alongside its unfavourable action on vascular endothelium, may contribute to rare thrombotic complications (both venous and arterial). Furthermore, EE can exert non-physiological effects on IGF1 signaling pathways by modifying IGF1 levels and the expression of IGF binding proteins, potentially impacting growth and development [18].

Vaginal or transdermal administration of EE also impacts hepatic proteins [19]. EE has been a component of many COCs commercially available for over 60 years.

## Effects of progesterone on bone tissue

Estradiol has always been recognized as the primary driver for bone health in women. However, substantial evidence also supports a role for progesterone.

Progesterone stimulates osteoblasts differentiation in vitro at concentrations comparable to luteal phase serum levels, independently of estradiol pre- or co-treatment. Conversely, supraphysiological doses suppress osteoblast differentiation [20]. Bone resorption markers are higher during the follicular phase of the menstrual cycle, while bone formation markers are higher during the luteal phase. A study in women aged 20–47 years during the luteal phase of the menstrual cycle demonstrated a correlation between serum progesterone levels and increased levels of osteocalcin, bone alkaline phosphatase (bALP), and insulin-like growth factor I (IGF-I) [21].

It is shown that in adolescent girls, bone mass gain is already significant after menarche but undergoes a marked acceleration with the onset of ovulatory cycles, which typically occurs about a year after menarche [20]. These findings suggest that progesterone, alongside estradiol, contributes to the attainment of peak bone mass during adolescence.

Premenopausal women with amenorrhea or oligomenorrhea, whether due to hypothalamic suppression (i.g. functional amenorrhea) or ovarian dysfunction (i.g. Turner syndrome or other causes), experience a decline in BMD driven by low levels of both estradiol and progesterone [22]. Some studies have shown that women aged 20–42 years with regular menstrual cycles and sufficient estrogens but subclinical ovulation disorders (anovulatory cycles or cycles with reduced luteal phase duration) may experience bone mass loss [23–27]. This demonstrates the role of progesterone in maintaining bone mass in premenopausal women. Additionally, a study showed that cyclic MPA treatment (10 mg/day on days 16–25) in women with subclinical ovulation disorders leads to bone mass gain after one year compared to significant bone mass loss in the placebo group [28].

A study in perimenopausal women (mean age 46 years), indicates that during ovulatory cycles (identified based on the plasma progesterone level threshold used to document ovulation), plasma bALP levels increase, while urinary pyridinoline levels decrease (turnover suppression is linked to the persistence of moderate estrogen levels during the luteal phase of the cycle) [20]. Another study in perimenopausal women (mean age 48 years) showed a direct correlation between anovulatory cycles and bone mass loss evaluated by QCT [29], demonstrating that the lack of progesterone secretion directly contributes to bone mass loss.

Furthermore, a study in women with surgical menopause (bilateral hysterectomy and oophorectomy) found that MPA therapy (10 mg/day) versus CEE (0.6 mg/day), initiated immediately after surgery, resulted in significant lumbar bone mass loss after one year of follow-up with both therapies (greater for MPA versus CEE) and failed to suppress bone turnover markers during MPA treatment. This demonstrates that postmenopausal progesterone therapy is not effective for bone health due to its lack of effect on bone resorption control [30].

Some studies have demonstrated that in postmenopausal women, therapy with MPA (10 mg/day) + micronized estradiol (1 mg/day) results in a significantly greater annual lumbar bone mass gain than estradiol monotherapy, highlighting the additive effect of progesterone to estradiol on bone health [31, 32].

A variety of progestogens are extensively used by women for both contraception and menopausal hormone therapy, and they can be categorized into three primary groups: pregnanes, gonanes, and estranes. These classifications are based on their chemical structures, which influence their specific pharmacological properties and clinical effects. All progestogens exert activity through binding to specific progesterone receptors; however, it is worth to note that each progestogen can exert distinct effects depending on its affinity for progesterone, glucocorticoid, mineralocorticoid, and androgen receptors, leading to vaying pharmacological properties and potentially diverse clinical outcomes [33]. The anabolic action of a progestogen can indeed be influenced by its androgenic, anti-androgenic, or synandrogenic activity [9].

## Effects of hormonal contraceptive therapies (HCT)

In hormonal contraception estrogen bioavailability varies depending on the type of formulations, whether it contains ethinyl estradiol, estradiol/estradiol valerate, estetrol, or is progestin-only (Table 1).

**Table 1:** Plasma Estradiol (E2) levels during different contraceptive use

Contraceptive type	Plasma E2 levels*	References
*Combined oral contraceptives (COCs) with ethinylestradiol*		
EE 30 µg/Levonorgestrel 150 mcg	E2: 20–40 pg/mL	[34]
EE 20 ug/Levonorgestrel 150 mcg	E2: 90 pg/ml	[35]
*Combined oral contraceptives (COCs) with estradiol*		
Estradiol 1.5 mg/Nomegestrol Acetate 2.5 mg	100 pg/mL	[36]
Estradiol Valerate 3-2-1 mg/Dienogest 2–3 mg	50–90 pg/mL	[37]
*Combined oral contraceptive (COC) with estretrol*		
E4 15 mg/Drospirenone 3 mg	30–40 pg/mL	[38]
*LNG IUS 13,5 mg, 19,5 mg, 52 mg*	Physiological levels	[39]
*Progestin-only contraceptives*		
POP with Dienogest 2 mg	30 pg/mL	[40]
POP with Levonorgestrel 30 µg	146 pg/mL	[41]
POP with Desogestrel 75 µg	73 pg/mL	[41]
POP with Drospirenone 4 mg	48 pg/mL	[42]
DMPA 150 mg IM (every 3 months)	25 pg/mL (ages 18–25) 35 pg/mL (ages 35–45)	[43]

^*^Plasma Estradiol (E2) levels are: (a) endogenous in COCs with ethinylestradiol/EE (with daily EE peaks up to 125 pg/ml; consider the biological activity of EE); (b) mixed exogenous and endogenous in COCs with Estradiol and Estradiol Valerato; (c) endogenous in COCs with Estetrol (consider the biological activity of Estetrol); (d) endogenous in POPs. DMPA, depomedroxyprogesterone acetate.

It has been known since the late 1970 s that the risk of thromboembolism correlates with the estrogen dose. Since then, combined hormonal contraceptives (CHCs) with decreasing doses of ethinylestradiol (EE) have been used, ensuring contraceptive efficacy and tolerance profile, partly due to the development of new progestogens. Moreover, since the first decade of the 2000 s, COCs containing natural estradiol have been marketed [16]. However, in the effort to ensure ever-greater safety regarding the pro-thrombotic profile, it is important to consider the comprehensive implications of these low and ultra-low estrogen dose formulations.

CHCs primarily function by inhibiting gonadotropin secretion through the hypothalamus, thereby preventing ovulation. Various formulations are available on the market, differing in the type and dosage of the estrogen component, the accompanying progestin, and the mode of delivery, such as pills, vaginal rings, or patches. Additionally, combined oral contraceptives (COCs) come in monophasic, biphasic, or triphasic formulations. Monophasic formulations maintain a constant dose of estrogen and progestin, but the regimen may vary in terms of the duration of hormonal discontinuation between cycles. These distinctions could account for varying effects on bone health.

Although existing evidence indicates that CHCs might affect bone turnover, the findings remain inconclusive. The intricate impact on bone is influenced by the specific biological effects of the estrogen used, alongside the dosage, the duration of hormonal exposure determined by the regimen, and the residual endogenous production of estradiol. A recent systematic review and meta-analysis [44] examined the effects of CHCs on bone metabolism markers in women of reproductive age. Overall, CHCs seem to either have a neutral effect or reduce bone turnover, but the extent of these effects varies depending on factors such as age and the specific combination of hormones involved. Across all age groups, CHCs containing weak estrogens and androgenic progestins are linked to a smaller reduction in bone turnover, potentially fostering a favorable balance between bone formation and resorption. CHCs with EE are particularly noted for reducing bone turnover, possibly causing an unfavorable balance between formation and resorption. Interestingly, the effect of EE is influenced by the associated progestin: it is most significant when paired with an anti-androgenic progestin and nearly negligible when combined with neutral or androgenic progestins [44]. Progestins with anti-androgenic activity lead to greater suppression of bone turnover through a decrease in osteocalcin levels compared to progestins with neutral or androgenic activity [44].

Although limited data are available on CHCs with natural estradiol, they suggest that during E2-CHC use resorption decreases more than formation, indicating a potentially positive effect on bone. Due to the scarcity of available data, limited conclusions can be drawn regarding the effects and implications of E4-based formulations.

Despite these findings, no significant changes in BMD have been observed with CHCs usage [45]. Long-term prospective studies are essential to further clarify the role of various estrogens and progestins on bone health throughout the reproductive stages of a woman’s life.

Progestin-only contraceptives are available as implantable capsules, intrauterine devices, oral minipills, and intramuscular or subcutaneous injections. The systemic dose of progestin delivered by these products is relatively low (except for the intramuscular depot medroxyprogesterone acetate (DMPA), with less suppression of endogenous estrogen levels [44]. The administration of depot medroxyprogesterone acetate (DMPA) 150 mg IM every 12 weeks is associated with a partially reversible significant decrease in lumbar and femoral BMD after 12 months of treatment compared to controls, who showed a significant increase in lumbar and femoral BMD [46]. Past studies show that in adolescents (12–18 years) and young women (18–25 years) E2 levels often fall below 30 pg/mL, leading to reductions in lumbar and femoral BMD. In contrast women aged 35–45 years, typically maintain E2 levels above 30 pg/mL with no adverse effects on BMD [44]. To date, no studies on injectable DMPA have evaluated fracture outcomes as a primary endpoint [46]. However, but some data suggest that women with more than 2 years of cumulative DMPA use may have a slightly increased fracture risk compared with other contraceptive methods [47]. Moreover, DMPA is a progestin with a strong glucocorticoid activity which directly and negatively affects bone turnover. However, it is no longer commonly used for contraceptive purpose.

Conversely, the potential for adverse skeletal effects from progestin-only contraceptives other than DMPA may be less significant. Progesterone-only pills (POPs) available in Italy with the indication for contraception contain drospirenone 4 mg tablet/day for 24 days and 4 hormone-free pills and desogestrel 75 mcg tablet/day for 28 days. POPs ensure endogenous E2 levels above 30 pg/mL and may have no impact on BMD when the user experiences normal ovarian activity and menstruation. Among lactating women, those using POPs, showed significantly less decline in BMD at 6 months and 1 year postpartum compared to non-users [9]. However, in hypoestrogenic situations the use of POPs may suppress the ovarian activity and increase the risk of low BMD [48].

Levonorgestrel-releasing intrauterine system (LNG-IUS) is also a widely used and efficient contraception method, with no significant adverse effects on BMD as well as on fracture risk [49, 50]: women during LNG-IUS use are generally ovulating with normal circulating E2 levels.

The impact of these therapies on bone health can differ across various stages of life and specific circumstances, as outlined in the following paragraphs.

### Effects of HCT in adolescence

Low-dose ethinyl-estradiol (EE 20 µg/day) COCs taken by eugonadal adolescents (12–19 years) for 12–24 months during the first three years post-menarche (a period dominated by bone formation) negatively impact peak bone mass attainment. Treated adolescents exhibit delayed and significantly lower bone mass acquisition compared to untreated peers due to insufficient estrogen replacement [51–54]. Conversely, supraphysiological EE doses (35 µg) can inhibit periosteal apposition and increase IGF-I binding protein 3 levels, reducing the beneficial effect of free IGF-I on bone [55]. Cibula et al. in their RCT on adolescents note that BMD of Lumbar Spine during adolescence may be interfered especially when ultra-low does COCs (with 15 μg of EE) are used [56]. Consequently, COCs containing medium dose of EE (30–35 µg) of EE are recommended for adolescents requiring hormonal contraception. Limited data are available on COCs with natural estradiol, and no studies report fracture outcomes. However, as previously discussed, E2-based COC may be more appropriate in conditions where bone formation is a primary concern, such as in cases involving pubertal girls [44]. Data on the short- and long-term changes in BMD with progestin-only contraceptives except for intramuscular DMPA in adolescents is limited [55]. However, there is some evidence that, in women ≤ 21 years of age, the administration of a progestin-only contraceptive did not reduce bone turnover and induced a positive balance between bone formation and reabsorption [44]. This suggests a less significant impact on skeletal outcomes compared to COCs in this subpopulation.

### Effects of HCT in polycystic ovarian syndrome

In PCOS, chronic anovulation leads to infrequent or absent corpus luteum formation and, consequently, insufficient progesterone secretion. This results in a state of unopposed estrogen action that reflects a relative, rather than absolute, estrogen excess. Women with PCOS frequently lack the normal cyclical fluctuations in estradiol; they may not exhibit the pre-ovulatory estradiol surge or the mid-luteal peak and instead maintain relatively stable levels. Nonetheless, circulating estradiol concentrations typically remain within the normal physiological range [56]. However, disturbances in estrogen physiology in PCOS may extend beyond absolute estradiol levels. The free (bioactive) fraction of estradiol appears to be increased, the estradiol-to-testosterone (E₂/T) ratio is reduced in oligo- or anovulatory women, and there is evidence of impaired estrogen signaling attributable to dysregulated receptor pathways [57–59]. Thus, the hormonal milieu in PCOS is highly complex; however, a detailed discussion of these mechanisms is beyond the scope of this review.

Conflicting data exist on the relationship between polycystic ovarian syndrome (PCOS) and bone [60, 61]. PCOS has been associated with a possible reduction in BMD, due to frequent anovulatory cycles and a reduced exposure to progesterone—which has a promoting effect on osteoblast proliferation—during anovulatory cycles [62]. Besides, it has been suggested that androgen excess, together with obesity and hyperinsulinism, may have a protective effect on bone in women with PCOS [63]. However, this effect seems to depend on maintaining a menstrual cycling, at least partially: in a study performed by Adami and colleagues [63], patients with PCOS-associated amenorrhea (defined as less than four menstrual cycles per year), had significantly lower spine and femoral neck BMD compared with subjects with non-amenorrheic PCOS.

Oral contraceptives are commonly prescribed to young women with menstrual alterations and PCOS, in order to regulate the menstrual cycle and to control hyperandrogenism in women with PCOS. Low dose ethinyl oestradiol COCPs are the preferred treatment for hirsutism in both adults and adolescents as they increase SHBG levels and decrease luteinizing hormone, serum testosterone and androstenedione levels [64].

As mentioned before, the use of COCs during adolescence, particularly oral formulations containing EE and low-dose (EE 20 mcg) or ultra-low-dose COCs (EE 15 mcg), negatively impact peak bone mass attainment [49, 65]. Consequently, with the aim of preserving bone mass, oral contraceptives with doses of EE greater than 30 μg formulations would be preferable to lower-dose preparations in adolescents [65]. However, PCOS-specific features, such as higher weight and cardiovascular risk factors, need to be considered, and guidelines still recommend lower dose estradiol (30 mcg or less) combined contraceptives to avoid possible adverse effects [64].

As previous reported [44], a higher reduction in bone turnover and a possible detrimental balance between formation and absorption is induced by COCs containing strong estrogens and anti-androgenic progestins, which are the preferred treatment in PCOS women to manage hirsutism.

On the other side, the reduction in androgen production obtained with COCs and anti-androgens may contribute to worsening the bone condition [66]. Some reports have documented the deleterious effects of antiandrogen therapy on bone mass in PCOS subjects [67], although available short-term data suggest no significant effect on bone loss [68].

In conclusion, more studies specifically designed in women suffering from polycystic ovary syndrome are needed to understand the impact of PCOS and its management, both in adolescence, when the impact could be more critical on bone, and in adulthood. A correct balance between the control of androgenic symptoms and the preservation of bone mass should be pursued.

### Effects of HCT in perimenopause

Bone loss begins during the “late” perimenopausal phase, characterized by amenorrhea lasting more than 3 months but less than 1 year. In this transitional phase, combined oral contraceptives (COCs) offer significant advantages beyond contraception, including menstrual cycle regulation, reduction in dysmenorrhea, alleviation of vasomotor symptoms, bone protection, and reduced risk of endometrial, ovarian, and colorectal cancer.

For bone health, low-dose COCs containing EE 20 µg in oligomenorrheic women significantly increase lumbar BMD, whereas untreated oligomenorrheic women experience significant BMD loss [69]. However, COCs containing estradiol or estradiol valerate are preferred, as they provide the same bone-protective effects with a lower thrombotic risk (arterial and venous) [70, 71].

Table 2 summarized the expected effect on BMD and/or bone turnover markers of different hormonal contraceptives, from adolescence to perimenopause.

**Table 2:** Expected effect on Bone Mineral Density (BMD) of different hormonal contraceptives, from adolescence to perimenopause

Contraceptive type	Effects on BMD	
*Progestin-only contraceptives*		
	<25 years old	>35 years old
POPs	Limited data, expected neutral*	
	*Decrease in hypoestrogenic conditions*	
LNG-IUS	*Neutral*	
	*Decrease in hypoestrogenic conditions*	
DMPA 150 mg IM (every 3 months)	*Decrease*	*Neutral*

	Adolescent	Childbearing potential	Perimenopause	
COCs with >35 µg EE	*Decrease*	*Neutral* May decrease if combined to anti-androgenic progestins* May increase/be neutral if combined to neutral or androgenic progestins*	*Increase*	
COCs with 30–35 µg EE	*Neutral*		*Increase*	
COCs with 15–20 µg EE	*Decrease*		*Increase*	
COCs with E2	Limited data, expected neutral*			Limited data, expected increase*
COCs with E4	*No data, expected neutral/increase*			

BMD: Bone Mineral Density; EE: ethinylestradiol; E2: estradiol; E4: estetrol; DMPA: depot medroxyprogesterone acetate; COCs: combined oral contraceptives; LNG-IUS: Levonorgestrel-releasing intrauterine system; POPs: Progestin-only pills

^*^Expected data derived from effect on Bone Turnover Markers (BTM)

## Treatment of symptomatic uterine fibroids in reproductive-age women

Since 2022, a combination therapy consisting of relugolix (oral GnRH antagonist) 40 mg, estradiol 1 mg, and norethisterone acetate 0.5 mg (doses for hormone replacement therapy) has been available for treating moderate-to-severe symptomatic uterine fibroids in reproductive-age women. The efficacy and safety of this treatment were evaluated in the LIBERTY 1 and LIBERTY 2 trials [72]. Relugolix combined with estrogen-progestin (EP) therapy was directly compared to placebo (and only indirectly to GnRH agonists), with cost-effectiveness estimates favouring relugolix + EP therapy.

This oral therapy, suitable for long-term use, offers an effective alternative to surgery, is well tolerated, and preserves the uterus and fertility. In phase 3 clinical trials, median pre-dose estradiol concentrations after 24 weeks of treatment were approximately 33 pg/mL, consistent with estradiol levels in the early follicular phase of the menstrual cycle. From a bone-health perspective, while GnRH analogue therapies suppress estradiol levels below 20 pg/mL, relugolix combined with EP therapy maintains estradiol levels between 20–50 pg/mL. This range is sufficient to preserve bone health while preventing menopausal-like symptoms.

The percentage changes in lumbar and femoral BMD from baseline to 12 and 24 weeks of treatment were similar in the group treated with relugolix + combination estrogen-progestin therapy and the placebo group. However, as expected, a decrease in lumbar and femoral BMD was observed in patients treated with relugolix alone for 12 weeks, with a plateau in the subsequent 12 weeks after the addition of combination therapy [72].

The National Institute for Health and Care Excellence (NICE) has issued guidelines for using this drug to treat uterine fibroids [73]. For bone health, it is recommended to perform a DXA scan before initiating treatment in patients with a history of fragility fractures or risk factors for osteoporosis or bone loss, including those on medications affecting BMD (e.g., corticosteroids). For high-risk patients, a follow-up DXA scan after 12 months is advised to ensure no excessive BMD loss outweighs the benefits of relugolix + EP therapy. Other drugs of the same family (oral GnRH antagonist), such as linzagolix, are also being launched for the treatment of fibroids and endometriosis, even in the absence of a hormonal add back therapy. According to E2 circulating levels, during therapy with relugolix 40 mg/estradiol 1 mg/norethisterone acetate 0.5 mg, they are exogenous, derived from the add back therapy, whereas they are endogenous during therapy with linzagolix alone [74].

## Effects of estro-progestin therapies in women with hypogonadism

Female hypogonadism (FH), whether secondary to central causes or due to primary ovarian insufficiency, results in amenorrhea accompanied by altered secretion of sex hormones, impaired ovarian folliculogenesis, and anovulation. It often presents with symptoms of hypoestrogenism and can occurs at any age from puberty to natural menopause. Clinically, FH has various implications for women’s health and overall well-being, and therefore, it requires accurate diagnosis and appropriate treatment. The causes of FH can be classified as either primary or central. Primary Ovarian Insufficiency (POI) is characterized by reduced or absent ovarian folliculogenesis, leading to insufficient secretion of sex hormones, typically presenting with low estradiol levels and elevated gonadotropin levels (FSH and LH). The causes of POI vary and may include genetic and chromosomal abnormalities (e.g. Turner Syndrome) or acquired causes (iatrogenic, autoimmune, environmental); however, it remains idiopathic in over 70% of cases [75]. Central or hypogonadotropic forms may be congenital, acquired (e.g., iatrogenic, tumor-related), or functional. Functional Hypothalamic Amenorrhea (FHA) is the most common form of functional CH, often triggered by relative energy deficiency (restricted nutritional intake and/or excessive physical exercise leading to low fat mass), psychosocial, or physical stress. Hormone replacement therapy (HRT) is mandatory in the absence of contraindications to address both short-term complications (vasomotor symptoms, mood alterations, sexual dysfunction) and long-term risks (cardiovascular disease, osteoporosis, cognitive impairment, and premature mortality) [76, 77].

Hormone preparations based on 17β-estradiol are favored for their physiological, safe, and effective profile, especially when administered transdermally. However, no formulations are specifically designed for the long-term treatment of young women with FH. Instead, therapies intended for postmenopausal climacteric symptoms or CHCs are commonly used. HRT regimen can be either combined sequential, introducing progesterone in the second phase of each cycle (for 12 to 14 days), or combined continuous, with progesterone taken at lower doses but throughout the entire month; however, treatment with 17β-estradiol should be continuous to avoid periods of hypoestrogenism. In women of childbearing age with FH, it is suggested that the dosage of HRT should be higher than that used in standard HRT for physiological menopause (age > 45 years) [78].

Women with FH exhibit significantly lower bone mineral density (BMD) and face a higher risk of fractures compared to those who experience menopause at the average age. This disparity is primarily due to insufficient attainment of peak bone mass, in cases of primary amenorrhea or early onset, and heightened bone resorption linked to estrogen deficiency. As far as bone health is concerned, HRT has been shown to maintain or increase bone mineral density (BMD), with effectiveness that seems to depend on the specific HRT regimen used.

A long-term, prospective, double-masked, controlled study demonstrated that a regimen of physiological HRT -comprising a 100 µg/day estradiol patch and 10 mg/day oral medroxyprogesterone acetate for 12 days per month- normalized BMD over three years in women with POI [79].

Studies comparing COCs (EE 30 µg) to physiologic HRT (estradiol 2 mg daily) have shown that both treatments similarly reduce bone turnover. However, after 2 years, lumbar BMD is maintained with COCs and increased with HRT, with both regimens outperforming no treatment [80]. Another study comparing COCs (EE 30 µg) with transdermal estradiol (100 µg daily for week 1, 150 µg daily for weeks 2–4) demonstrated that lumbar BMD is maintained with COCs and increased with hormone replacement therapy (HRT) after 12 months. Regarding bone turnover markers, both therapies suppressed serum C-terminal telopeptides, but only HRT increased bone formation markers (bone-specific isoenzyme of alkaline phosphatase and pro-collagen I N-terminal propeptide) after 12 months [81]. A systematic review and meta-analysis on the effect of HRT on bone outcomes in women with Turner syndrome showed a significant increase in BMD of the lumbar spine with the use of HRT; the increase in BMD was greater with the use of 17-beta-estradiol compared with synthetic estrogen [82, 83]. However, a meta-analysis that directly compared the effect of COCs and physiological HRT on BMD in women with POI presents inconclusive results, mainly due to the limitations of existing studies. It should be noted that it was observed that studies in favour of HRT over COCs for BMD used regimens of non-continuous COCs [81]. Regarding therapeutic doses, although standard therapy appears to maintain stable BMD, there are indications that higher doses or continuous estrogen exposure regimens (including continuous COCs) may have a more significant impact, potentially improving BMD [84]. However, the data are not conclusive in this regard [85]. Another controversial issue is the route of administration: most of the evidence is still inclusive, however, some data suggest that transdermal estradiol shown a more favorable impact on BMD compared to oral estradiol in girls with Turner Syndrome [86]. It should be noted that most of the data have been obtained in the population of women with POI, particularly women with Turner syndrome. Importantly, distinct clinical and biological features within the Turner syndrome population preclude it from being fully comparable to other etiologies of hypogonadism.

## Effects of estro-progestin therapies in women with Turner Syndrome

In the specific context of Turner Syndrome, the increased risk of fragility fractures results from multiple factors beyond estrogen deficiency, including chromosomal abnormalities and gene haploinsufficiency, nutritional deficiencies related to associated celiac disease and inflammatory bowel disease, as well as a higher risk of falls due to impaired hearing, balance and spatial coordination [77]. Moreover, TS is characterized by reduced areal and volumetric BMD, particularly affecting cortical bone, while trabecular bone is often relatively preserved [87]. Early initiation and continuous use of HRT is a cornerstone of managing bone health and is associated with better bone quality and density [88]. Indeed, beyond the psychosocial benefit of entering puberty alongside peers, initiating pubertal induction with HRT at a physiological age (around 11 years) supports normal bone mineral accrual. To optimize bone health in adulthood it is suggest monitoring serum E2 concentrations, with the goal to achieve E2 concentrations of 100–150 pg/mL (350–500 pmol/L) at full adult replacement [89]. As mentioned, studies suggest that 17β-estradiol has a more favorable effect on BMD than synthetic estrogens [83], and that transdermal estradiol is superior to oral formulations [86]. Therefore, physiologic HRT delivered transdermally may be preferred to optimize bone health [89].

## Effects of estro-progestin therapies in women with functional hypothalamic amenorrhea (FHA)

Central hypogonadotropic (CH) forms may be congenital, acquired (e.g., iatrogenic, tumor-related), or functional. FHA is the most common form of functional CH, often triggered by relative energy deficiency (restricted nutritional intake and/or excessive physical exercise leading to low fat mass), psychosocial, or physical stress. This condition is particularly evident in the context of the female athlete triad, a syndrome characterized by the interrelationship among decreased energy availability, menstrual dysfunction, and low bone density. Low energy availability appears to be the factor that impairs reproductive and skeletal health, both directly via metabolic hormones (such as insulin, cortisol, growth hormone, IGF-I, thyroid hormones and leptin) and indirectly via effects on estrogens’ production [90]. Athletes with amenorrhea, in comparison to eumenorrheic athletes, have lower levels of leptin, thyroid hormones, FSH, estradiol, progesterone, and insulin, along with elevated ghrelin and cortisol levels and a reduced ratio of IGF-I to IGFBP-I (a binding protein of IGF-I), indicating less bioavailable IGF-I [91]. Moreover, excessive exercise, particularly when energy availability is inadequate, is associated with negative effects on bone [91].

Notably, the meta-analysis by Aalberg et al. focuses on the effect of estrogen therapy on BMD in premenopausal women with FHA [92]. The results of the meta-analysis, when considered as a whole, revealed no statistically significant differences in bone mineral density (BMD) between the study groups receiving estrogen therapy (including COCs, CEEs, and transdermal estradiol [TE]) and the control groups. In contrast, two RCTs [93, 94] employing the transdermal estradiol patch in girls with FHA demonstrated a substantial increase in spinal BMD in the treated cohort compared to the placebo group and the group treated with EE. The authors posit that a potential explanation for the observed discrepancy between the efficacy of oral and transdermal therapies may be attributed to the hepatic "first-pass" metabolism of oral estrogens. It is postulated that this metabolic process, particularly in the case of synthetic estrogens, may result in a reduction in levels of IGF-1, a hormone that plays a pivotal role in bone growth. This phenomenon is of relevance to patients with FHA, as their low baseline levels of IGF-1 make them particularly susceptible to this potential effect [92]. Anyhow, the first aim of treatment for FHA in the context of female athlete triad is to increase energy availability by increasing energy intake and/or reducing exercise energy expenditure [90]. The use of COCs to restore menstrual cycles is not recommended, as they may cover a possible spontaneous physiological recovery and provide a false sense of reassurance, with limited to no benefit on BMD. In case of failure in reestablishing menstruation after 6 to 12 months of nutritional, psychological and exercise-related interventions associated with a proven decrease in BMD, the suggested approach consists of transdermal estradiol therapy associated with a cyclic oral progestin; however, bone health may not be protected with E2 replacement therapy if nutritional factors/energy deficiency continue [77, 95, 96]. Moreover, the need for contraception may also be an important consideration.

In conclusion, HRT is certainly essential in maintaining adequate bone mass in women with FH, however, there is still a lack of comprehensive, well-designed studies specifically addressing the optimal HRT regimen for bone protection according to different clinical settings.

## Effects of estro-progestin therapies in postmenopause

In postmenopause, HRT is indicated for symptomatic women with vasomotor disturbances who are <60 years old and within 10 years of menopause onset (last menstrual period). In women aged 50–59 years without symptoms of perimenopause/menopause, HRT could be considered as one of the first-line therapies for the prevention of postmenopausal osteoporosis and related fractures in postmenopausal women at increased fracture risk and younger than 60 years, or within 10 years of menopause [97, 98]. The Endocrine Society Guidelines published in 2020 for the treatment of postmenopausal osteoporosis recommend considering HRT or selective estrogen receptor modulator (SERMs) in women eligible for anti-resorptive therapy but intolerant or unsuitable for bisphosphonates or denosumab [99].

HRT and SERMs provide potent bone-protective effects and significantly reduce fracture risk. Specifically, HRT reduces all fracture types (vertebral, non-vertebral, and femoral), tibolone reduces vertebral and non-vertebral fractures but not hip fractures, SERMs (raloxifene and bazedoxifene) reduce vertebral fractures only [98, 100]. Use of HRT should be considered as a first therapeutic option to prevent osteoporosis in early postmenopausal women with a low to moderate risk of fracture where specific bone active medications may not be appropriate, and the initial part of a longer-term management strategy [100, 101]. Oral and transdermal oestrogen therapies appear to have similar effects on BMD, with lesser improvement observed with lower-than-standard doses of oestrogen compared to standard dose [102]. Subsequent reassessment of the individual benefit-risk balance is recommended, with the possibility of switching to another osteoporosis treatment—or alternatively of combining HT with a bone active drug—if the fracture risk remains high [100]. The most recent European Society of Endocrinology Guidelines published in 2025 for the management of menopause highlighted that HRT is not considered a first treatment for postmenopausal women with established osteoporosis and that bone specific agents are recommended as first line treatment in this setting [98].

The discontinuation of HRT/SERM in post-menopausal women determines an increase in bone turnover, a decrease in BMD and a reduction in anti-fracture protection [101], therefore in women over 65 years of age and with low BMD sequential therapy with an anti-resorptive drug can counteract this rebound effect [103].

## Effects of off-label testosterone

Hypoactive Sexual Desire Disorder (HSDD) is defined as a lack of motivation for sexual activity, manifested by a reduction or absence of spontaneous sexual desire (sexual thoughts or fantasies) or of desire responsive to erotic stimuli, or by an inability to maintain desire during sexual activity for a period of at least six months. This decline in sexual desire is not secondary to sexual pain and is associated with clinically significant personal distress. Women with HSDD in whom inhibitory factors predominate appear to exhibit greater self-control and a higher tendency toward self-evaluation and self-criticism [104]. In double-blind, placebo-controlled clinical trials in naturally and surgically menopausal women, testosterone therapy resulted in statistically significant improvements in the number of satisfying sexual events, sexual desire, and sexual distress that were two-fold greater than placebo [105].

Data about the effects of exogenous testosterone on bone in women are scant. The available studies concerning the effects of exogenous testosterone on bone health are limited, involving small patient series and generally short follow-up periods. Findings regarding BMD are inconsistent: some studies report an improvement in BMD with combined estrogen–progestin and testosterone therapy compared to estrogen–progestin therapy alone, whereas others do not demonstrate an additive effect. No data on fracture outcomes are currently available [106–110].

## Conclusions

Maintaining bone health throughout a woman’s lifespan, especially during periods of increased vulnerability to bone loss, is a key consideration when selecting estrogen-progestin therapies. Evidence indicates that combined oral contraceptives generally do not adversely affect BMD in adult women, but may hinder optimal BMD acquisition during adolescence, particularly with very low-dose estrogen formulations (Table 3).

**Table 3:** Estrogen-progestin therapies ensuring bone health according to woman’s age

Age	Estrogen-progestin therapy
Adolescence	COCs containing EE 30–35 µg
Premenopausal Adults	COCs containing EE 20–35 µg, estradiol/estradiol valerate, or estetrol, and progestin-only pills
Perimenopause	COCs containing estradiol or estradiol valerate
Premature Ovarian Insufficiency (POI)	Hormone replacement therapy
Postmenopause	Postmenopausal Hormone therapy

COCs, combines oral contraceptives; EE, ethynilestradiol

In conditions of female hypogonadism, where estrogen deficiency significantly impacts bone health, hormone replacement therapy (HRT) is critical for reducing short- and long-term complications, including osteoporosis. Although the optimal therapeutic regimen has not yet been clearly established, physiological regimens, preferably using transdermal estrogen and at doses higher than those typically used in postmenopausal therapy, may be recommended to optimize bone protection. Furthermore, therapies such as oral GnRH antagonists combined with add-back therapies offer a novel approach for managing uterine fibroids and endometriosis while maintaining bone mineral density and preventing menopausal symptoms.

In postmenopause, HRT and selective estrogen receptor modulators (SERMs) provide significant fracture risk reduction and overall bone protection. These therapies also offer broader systemic benefits, including vasomotor symptom control and cardiovascular protection. Future research should focus on optimizing therapeutic regimens to balance efficacy and safety, ensuring personalized approaches that maximize both bone health and quality of life for women across lifespan.
