# Correction: Impact of estroprogestin therapies on bone health from adolescence to postmenopause

**DOI:** 10.1007/s40618-026-02863-x

**Published:** 2026-04-28

**Authors:** Guabello Gregorio, Federici Silvia, Carrara Silvia, Ruotolo Elena, Rossi Antonio, Bertini Jacopo, Bonomi Marco, Grandi Giovanni, Corbetta S.

**Affiliations:** 1 Division of Internal Medicine, Endocrinology and Metabolic Diseases, I.R.C.C.S. Ospedale Galeazzi – Sant’Ambrogio, Milan, Italy; 2 Department of Endocrine and Metabolic Diseases, I.R.C.C.S. Istituto Auxologico Italiano, Milan, Italy; 3 Department of Medical Biotechnology and Translational Medicine, University of Milan, Milan, Italy; 4 Department of Biomedical and Clinical Sciences, University of Milan, Milan, Italy; 5 Department of Medical and Surgical Sciences for Mother, Child and Adult, University of Modena and Reggio Emilia, Modena, Italy; 6 Azienda Ospedaliero Universitaria, Modena, Italy; 7 Bone Metabolism Diseases and Diabetes Unit, IRCCS Istituto Auxologico Italiano, Via L. Ariosto 9, 20145 Milan, Italy; 8 Department of Biomedical, Surgical and Dental Sciences, University of Milan, Milan, Italy

**Correction: Journal of Endocrinological Investigation**

10.1007/s40618-025-02802-2

In the original version of this article, the given and family names of Sabrina Corbetta were incorrectly structured. The name was displayed correctly in all versions at the time of publication.

The original article has been corrected.

